# Supplementary material for: Accelerated Resolution Therapy (ART) for the treatment of posttraumatic stress disorder in adults: A systematic review
Source: PLOS Ment Health. 2024 Sep 17;1(4):e0000123. doi: 10.1371/journal.pmen.0000123 (PMC12798211; doi:10.1371/journal.pmen.0000123)
Supplement: S2 Appendix — (PDF) [file pmen.0000123.s002.pdf]

S2 Appendix. Search terms.

Databases

PsycInfo
(MM "Posttraumatic Stress Disorder" OR ptsd or "post traumatic stress disorder" or "posttraumatic stress disorder" or "post-traumatic stress disorder") AND "Accelerated Resolution Therapy"

Embase
('posttraumatic stress disorder/exp' OR ptsd or "post traumatic stress disorder" or "posttraumatic stress disorder" or "post-traumatic stress disorder") AND 'accelerated resolution therapy'

PubMed
(Accelerated Resolution Therapy) AND ("Stress Disorders, Post Traumatic"[Mesh] OR ptsd or "post traumatic stress disorder" or "posttraumatic stress disorder" or "post-traumatic stress disorder"))

CINAHL
("(MH "Stress Disorders, Post-Traumatic+") or ptsd or post traumatic stress disorder or posttraumatic stress disorder or post-traumatic stress disorder") AND "accelerated resolution therapy"

Scopus
(ALL ("accelerated resolution therapy")) AND ALL ("posttraumatic stress disorder" OR ptsd OR "post traumatic stress disorder" OR "post-traumatic stress disorder")

Registers

Cochrane Library Trials Database
("posttraumtic stress disorder" OR ptsd or "post traumatic stress disorder" or "posttraumatic stress disorder" or "post-traumatic stress disorder") AND "accelerated resolution therapy" in All Text

ClinicalTrials.gov
Condition or disease: posttraumatic stress disorder; *Other terms:* accelerated resolution therapy

WHO ICTRP
Intervention: accelerated resolution therapy

Organization Websites

U.S. Department of Veterans Affairs (VA) *https://www.va.gov/*
"Accelerated resolution therapy"

APA Division 12 *https://div12.org/diagnosis/posttraumatic-stress-disorder/*
Clinical trials and studies listed for Accelerated Resolution Therapy for Posttraumatic Stress Disorder

ART *https://acceleratedresolutiontherapy.com/evidence-based/*
Publications and case studies listed under: About ART > Evidence Based
