# Supplementary material for: Accelerated Resolution Therapy (ART) for the treatment of posttraumatic stress disorder in adults: A systematic review
Source: PLOS Ment Health. 2024 Sep 17;1(4):e0000123. doi: 10.1371/journal.pmen.0000123 (PMC12798211; doi:10.1371/journal.pmen.0000123)
Supplement: S3 Appendix — (PDF) [file pmen.0000123.s003.pdf]

S3 Appendix. Missing information requested, author contact details, and response rate from authors contacted Oct/Noc 2023.

Missing Information

Primary Studies<br>Reports of Studies	Summary statistics (*M, SD, n*) NOT reported for every outcome by group (if appl) at every time-point	Within & between (if appl) measures of effect with 95% CI's NOT reported for every outcome at every time-point	Important components of methodology not reported (e.g., power calculation, method of randomization, etc.)
**Kip et al. (2012)**		X	X
**Kip, Rosenzweig et al. (2013)**	X	X	X
**Kip et al. (2016)**	X	X	X
**Buck et al. (2020)**	X	X	
**Rossiter et al. (2017)**	X	X	X
**Kip, Sullivan, et al. (2013)**			
**Witt (2019)**	X	X	
**Pang et al. (2021)**	X	X	
**Kip et al. (2015)**	X	X	
**Hardwick (2017)**	X	X	X
**Kip et al. (2019)**	X	X	

Author

Corresponding Authors	Contacted via institutional email listed in publication	# of Tries	Contacted via email obtained through search or referral	# of Tries	Contacted via LinkedIn	# of Tries	Contacted via private practice webpage/phone	# of Tries
**Dr. Kevin E. Kip**	**X** (~~kkip@health.usf.edu~~)	1	**X** (kipke2@upmc.edu)	1	**X**	1		
**Dr. Harleah G. Buck**	**X** (~~hgbuck@usf.edu~~)	1	**X** (harleah-buck@uiowa.edu)	2				
**Dr. Alicia G. Rossiter**	**X**(arossite@health.usf.edu)	3						
**Dr. Ann Witt**	Email not listed						**X** (Private Practice DM/#)	3
**Dr. Tiantian Pang**	Email not listed		**X** (tiantianp@usf.edu)[a]	2				
**Dr. Marian J. Hardwick**	Email not listed				**X**	1		

Respon

No Response to Request	Unable to Fulfill Request	Partial Fulfillment of Request	Fulfillment of Request
**Dr. Alicia G. Rossiter**<br>**Dr. Ann Witt**<br>**Dr. Marian J Hardwick**		Dr. Kevin E. Kip [b, c]<br>Dr. Harleah G. Buck [b]<br>Dr. Tiantian Pang [b]	

[a] Recently graduated from USF; institutional email will be out of service by time of review publication.

[b] No longer works at the University of South Florida and does not have access to study data to provide missing summary statistics or effect sizes.

[c] Partially fulfilled request for information on Hardwick (2017), as he was her dissertation chair.
