# Supplementary material for: Accelerated Resolution Therapy (ART) for the treatment of posttraumatic stress disorder in adults: A systematic review
Source: PLOS Ment Health. 2024 Sep 17;1(4):e0000123. doi: 10.1371/journal.pmen.0000123 (PMC12798211; doi:10.1371/journal.pmen.0000123)
Supplement: S4 Appendix — (PDF) [file pmen.0000123.s004.pdf]

S4 Appendix. NIH study quality assessment checklists.

Kip et al. (2012). Brief Treatment of Symptoms of Post-Traumatic Stress Disorder (PTSD) by Use of Accelerated Resolution Therapy (ART)

Quality Assessment Tool for Observational Cohort and Cross-Sectional Studies

Criteria	Yes	No	Other (CD, NR, NA)*
1. Was the research question or objective in this paper clearly stated?		X	
2. Was the study population clearly specified and defined?	X		
3. Was the participation rate of eligible persons at least 50%?	X		
4. Were all the subjects selected or recruited from the same or similar populations (including the same time period)? Were inclusion and exclusion criteria for being in the study prespecified and applied uniformly to all participants?	X		
5. Was a sample size justification, power description, or variance and effect estimates provided?		X	
6. For the analyses in this paper, were the exposure(s) of interest measured prior to the outcome(s) being measured?	X		
7. Was the timeframe sufficient so that one could reasonably expect to see an association between exposure and outcome if it existed?	X		
8. For exposures that can vary in amount or level, did the study examine different levels of the exposure as related to the outcome (e.g., categories of exposure, or exposure measured as continuous variable)?	X		
9. Were the exposure measures (independent variables) clearly defined, valid, reliable, and implemented consistently across all study participants?	X		
10. Was the exposure(s) assessed more than once over time?	X		
11. Were the outcome measures (dependent variables) clearly defined, valid, reliable, and implemented consistently across all study participants?	X		
12. Were the outcome assessors blinded to the exposure status of participants?			NA
13. Was loss to follow-up after baseline 20% or less?		X	
14. Were key potential confounding variables measured and adjusted statistically for their impact on the relationship between exposure(s) and outcome(s)?	X		
Quality Rating: **Fair**			
Rater #1 initials: EM			
Rater #2 initials: DS			
Additional Comments (If POOR, please state why):			

*CD, cannot determine; NA, not applicable; NR, not reported

Kip et al. (2013). Randomized Controlled Trial of Accelerated Resolution Therapy (ART) for Symptoms of Combat-Related Post-Traumatic Stress Disorder (PTSD)

Quality Assessment of Controlled Intervention Studies

Criteria	Yes	No	Other (CD, NR, NA)*
1. Was the study described as randomized, a randomized trial, a randomized clinical trial, or an RCT?	X		
2. Was the method of randomization adequate (i.e., use of randomly generated assignment)?	X		
3. Was the treatment allocation concealed (so that assignments could not be predicted)?	X		
4. Were study participants and providers blinded to treatment group assignment?		X	
5. Were the people assessing the outcomes blinded to the participants' group assignments?			NA
6. Were the groups similar at baseline on important characteristics that could affect outcomes (e.g., demographics, risk factors, co-morbid conditions)?	X		
7. Was the overall drop-out rate from the study at endpoint 20% or lower of the number allocated to treatment?		X	
8. Was the differential drop-out rate (between treatment groups) at endpoint 15 percentage points or lower?	X		
9. Was there high adherence to the intervention protocols for each treatment group?	X		
10. Were other interventions avoided or similar in the groups (e.g., similar background treatments)?	X		
11. Were outcomes assessed using valid and reliable measures, implemented consistently across all study participants?	X		
12. Did the authors report that the sample size was sufficiently large to be able to detect a difference in the main outcome between groups with at least 80% power?	X		
13. Were outcomes reported or subgroups analyzed prespecified (i.e., identified before analyses were conducted)?	X		
14. Were all randomized participants analyzed in the group to which they were originally assigned, i.e., did they use an intention-to-treat analysis?	X		
Quality Rating: **Good**			
Rater #1 initials: EM			
Rater #2 initials: DS			
Additional Comments (If POOR, please state why):			

*CD, cannot determine; NA, not applicable; NR, not reported

Kip et al. (2016). Evaluation of brief treatment of symptoms of psychological trauma among Veterans residing in a homeless shelter by use of Accelerated Resolution Therapy

Quality Assessment Tool for Observational Cohort and Cross-Sectional Studies

Criteria	Yes	No	Other (CD, NR, NA)*
1. Was the research question or objective in this paper clearly stated?	X		
2. Was the study population clearly specified and defined?	X		
3. Was the participation rate of eligible persons at least 50%?	X		
4. Were all the subjects selected or recruited from the same or similar populations (including the same time period)? Were inclusion and exclusion criteria for being in the study prespecified and applied uniformly to all participants?	X		
5. Was a sample size justification, power description, or variance and effect estimates provided?		X	
6. For the analyses in this paper, were the exposure(s) of interest measured prior to the outcome(s) being measured?	X		
7. Was the timeframe sufficient so that one could reasonably expect to see an association between exposure and outcome if it existed?	X		
8. For exposures that can vary in amount or level, did the study examine different levels of the exposure as related to the outcome (e.g., categories of exposure, or exposure measured as continuous variable)?	X		
9. Were the exposure measures (independent variables) clearly defined, valid, reliable, and implemented consistently across all study participants?	X		
10. Was the exposure(s) assessed more than once over time?	X		
11. Were the outcome measures (dependent variables) clearly defined, valid, reliable, and implemented consistently across all study participants?	X		
12. Were the outcome assessors blinded to the exposure status of participants?			NA
13. Was loss to follow-up after baseline 20% or less?		X	
14. Were key potential confounding variables measured and adjusted statistically for their impact on the relationship between exposure(s) and outcome(s)?	X		
Quality Rating: **Fair**			
Rater #1 initials: EM			
Rater #2 initials: DS			
Additional Comments (If POOR, please state why):			

*CD, cannot determine; NA, not applicable; NR, not reported

Buck et al. (2020). Accelerated Resolution Therapy: Randomized Controlled Trial of a Complicated Grief Intervention

Quality Assessment of Controlled Intervention Studies

Criteria	Yes	No	Other (CD, NR, NA)*
1. Was the study described as randomized, a randomized trial, a randomized clinical trial, or an RCT?	X		
2. Was the method of randomization adequate (i.e., use of randomly generated assignment)?	X		Block randomization confirmed by author
3. Was the treatment allocation concealed (so that assignments could not be predicted)?			NR
4. Were study participants and providers blinded to treatment group assignment?		X	
5. Were the people assessing the outcomes blinded to the participants' group assignments?			NA
6. Were the groups similar at baseline on important characteristics that could affect outcomes (e.g., demographics, risk factors, comorbidities)?	X		
7. Was the overall drop-out rate from the study at endpoint 20% or lower of the number allocated to treatment?	X		
8. Was the differential drop-out rate (between treatment groups) at endpoint 15 percentage points or lower?		X	
9. Was there high adherence to the intervention protocols for each treatment group?	X		
10. Were other interventions avoided or similar in the groups (e.g., similar background treatments)?	X		
11. Were outcomes assessed using valid and reliable measures, implemented consistently across all study participants?	X		
12. Did the authors report that the sample size was sufficiently large to be able to detect a difference in the main outcome between groups with at least 80% power?	X		
13. Were outcomes reported or subgroups analyzed prespecified (i.e., identified before analyses were conducted)?	X		
14. Were all randomized participants analyzed in the group to which they were originally assigned, i.e., did they use an intention-to-treat analysis?	X		
Quality Rating: **Poor**			
Rater #1 initials: EM			
Rater #2 initials: DS			
Additional Comments (If POOR, please state why):<br><br>The differential drop-out rate between treatment groups at the endpoint of the study was 27% which is greater than the 15% cap set by the NIH. This represents a serious potential for bias and thus constitutes a fatal flaw, resulting in a poor-quality rating for the study despite its other merits.			

*CD, cannot determine; NA, not applicable; NR, not reported

Rossiter et al. (2017). Accelerated Resolution Therapy for Women Veterans Experiencing Military Sexual Trauma Related Post Traumatic Stress Disorder

Quality Assessment Tool for Before-After (Pre-Post) Studies With No Control Group

Criteria	Yes	No	Other (CD, NR, NA)*
1. Was the study question or objective clearly stated?	X		
2. Were eligibility/selection criteria for the study population prespecified and clearly described?	X		
3. Were the participants in the study representative of those who would be eligible for the test/service/intervention in the general or clinical population of interest?	X		
4. Were all eligible participants that met the prespecified entry criteria enrolled?	X		
5. Was the sample size sufficiently large to provide confidence in the findings?		X	
6. Was the test/service/intervention clearly described and delivered consistently across the study population?	X		
7. Were the outcome measures prespecified, clearly defined, valid, reliable, and assessed consistently across all study participants?	X		
8. Were the people assessing the outcomes blinded to the participants' exposures/interventions?			NA
9. Was the loss to follow-up after baseline 20% or less? *(Yes)* Were those lost to follow-up accounted for in the analysis? *(No)*		X	
10. Did the statistical methods examine changes in outcome measures from before to after the intervention? Were statistical tests done that provided p values for the pre-to-post changes?	X		
11. Were outcome measures of interest taken multiple times before the intervention and multiple times after the intervention (i.e., did they use an interrupted time-series design)?		X	
12. If the intervention was conducted at a group level (e.g., a whole hospital, a community, etc.) did the statistical analysis take into account the use of individual-level data to determine effects at the group level?			NA
Quality Rating: **Poor**			
Rater #1 Initials: EM			
Rater #2 Initials: DS			
Additional Comments (If POOR, please state why):<br><br>This article is missing crucial information needed to determine the methodological quality of the study. Some potential flaws include the small sample size, the lack of information provided about recruitment, and not accounting for those lost to follow-up in the analysis.			

*CD, cannot determine; NA, not applicable; NR, not reported
