# Supplementary material for: Accelerated Resolution Therapy (ART) for the treatment of posttraumatic stress disorder in adults: A systematic review
Source: PLOS Ment Health. 2024 Sep 17;1(4):e0000123. doi: 10.1371/journal.pmen.0000123 (PMC12798211; doi:10.1371/journal.pmen.0000123)
Supplement: S6 Appendix — (PDF) [file pmen.0000123.s006.pdf]

S6 Appendix. Reasons for excluding studies or reports of studies during full text screening.

#	Author (Year)	Title	Included?	Reason for Exclusion (if applicable)
1	Kip, et al. (2012)	Brief Treatment of Symptoms of Post-Traumatic Stress Disorder (PTSD) by Use of Accelerated Resolution Therapy (ART)	Yes	N/A
2	Kip, Sullivan, et al. (2013)	Brief treatment of co-occurring post-traumatic stress and depressive symptoms by use of accelerated resolution therapy	Yes	N/A
3	Kip, Rosenzweig, et al. (2013)	Randomized controlled trial of Accelerated Resolution Therapy (ART) for symptoms of combat-related post-traumatic stress disorder (PTSD)	Yes	N/A
4	Kip, Shuman, et al. (2014)	Case report and theoretical description of accelerated resolution therapy (ART) for military-related post-traumatic stress disorder	No	Wrong outcomes—no unique outcome data presented for the case
5	Kip, Rosenzweig, et al. (2014)	Accelerated Resolution Therapy for treatment of pain secondary to symptoms of combat-related posttraumatic stress disorder	No	Wrong outcomes—no unique outcome data presented for symptoms of PTSD
6	Kip et al. (2015)	Comparison of accelerated resolution therapy (ART) for treatment of symptoms of PTSD and sexual trauma between civilian and military adults	Yes	N/A
7	Kip et al. (2016)	Evaluation of brief treatment of symptoms of psychological trauma among veterans residing in a homeless shelter by use of Accelerated Resolution Therapy	Yes	N/A
8	Rossiter et al. (2017)	Accelerated Resolution Therapy for women veterans experiencing military sexual trauma related post-traumatic stress disorder	Yes	N/A
9	Hardwick (2017)	Examination of the use of accelerated resolution therapy (ART) in the treatment of symptoms of PTSD and sleep dysfunction in veterans and civilians	Yes	N/A
10	Witt (2019)	Predictors of veteran PTSD symptom reduction by use of accelerated resolution therapy	Yes	N/A
11	Kip et al. (2019)	The emergence of accelerated resolution therapy for treatment of post-traumatic stress disorder: A review and new subgroup analyses	Yes	N/A

12	Toukolehto et al. (2020)	Accelerated Resolution Therapy-Based Intervention in the Treatment of Acute Stress Reactions During Deployed Military Operations	No	Wrong intervention—ART was modified from established protocols, and not administered consecutively in all cases
13	Buck et al. (2020)	Accelerated resolution therapy: Randomized controlled trial of a complicated grief intervention	Yes	N/A
14	Pang et al. (2021)	Comparison of Accelerated Resolution Therapy for PTSD Between Veterans With and Without Prior PTSD Treatment	Yes	N/A
15	Tofthagen et al. (2022)	Accelerated Resolution Therapy for cancer related trauma and distress: a pilot study	No	Wrong indication—specific to cancer related distress

Note. Hardwick (2017) and Witt (2019) are unpublished dissertations, copies of which can be accessed using the embedded hyperlinks.
