# Supplementary material for: Accelerated Resolution Therapy (ART) for the treatment of posttraumatic stress disorder in adults: A systematic review
Source: PLOS Ment Health. 2024 Sep 17;1(4):e0000123. doi: 10.1371/journal.pmen.0000123 (PMC12798211; doi:10.1371/journal.pmen.0000123)
Supplement: S1 Table — (PDF) [file pmen.0000123.s007.pdf]

S1 Table. Characteristics of reports of studies included in the systematic review of ART for the treatment of PTSD in adults.

Author (Year)	Quality Rating[a]	Study Design	Population	Attrition[b]	Inclusion Criteria	Intervention Details	Comparison Group	Treatment Setting	Adverse Events	Funding Source
**Observational Trials & Studies**										
**2013-2015 – Registered Clinical Trial (NCT02030522): Prospective Cohort Study of Accelerated Resolution Therapy for the Treatment of Military Psychological Trauma ($n_{enrolled}$[c] = 140† US service members)**										
**Witt (2019)**	NA	Dissertation/Secondary – sub-group analysis: *# of deployments, guilt, depression, & anxiety; PTSD symptom severity*	veterans, $n_{completed}$[d] = 108 [M_{Age} = 43, 7% female, 85% white]	NR	≥18yrs; PCL-M ≥ 40, or PDSQ PTSD subscale ≥ 5; *(Low severity: PCL-M = 40-50, Mod severity: PCL-M = 51-60, High severity: PCL-M ≥ 61)*	2-5x (*M* = NR) 60-75min sessions	NA	NA	NA	NR
**Pang et al. (2021)**	NA	Secondary – sub-group analysis: prior PTSD Tx[e]	veterans, $n_{completed}$ = 106 [M_{Age} = 44, 5% female, 84% white]	42/148 (28%) 55/106 (48%) 97/148 (66%)	≥18yrs; PCL-M ≥ 40, or PDSQ PTSD subscale ≥ 5	1-5x (*M* = 3.5) 60-75min sessions	NA	NA	NA	DoD; Chris T. Sullivan Foundation
**2012 – Unregistered Study (Kip et al.): Brief Treatment of Symptoms of Post-Traumatic Stress Disorder by Use of Accelerated Resolution Therapy ($n_{enrolled}$ = 80 mostly civilians)**										
**Kip, Sullivan, et al. (2013)**	NA	Secondary – sub-group analysis: *comorbid PTSD & depression*	Adults[f] $n_{enrolled}$ = 35; $n_{completed}$ = 28 [M_{Age} = 41, 79% female, 93% white]	4/35 (11%) 3/31 (10%) 7/35 (20%)	21-60yrs; PCL-C ≥ 44, or PDSQ PTSD subscale ≥ 5; PDSQ items 20 & 21; CES-D ≥ 16, and PDSQ MDD subscale ≥ 9	1-5x (*M* = 3.7) 60-75min sessions	NA	NA	NA	SAMHSA; DoD
**Pooled Data from Kip et al., 2012 ($n_{enrolled}$ = 80 mostly civilians) and Registered Clinical Trial NCT01559688 ($n_{enrolled}$ = 57 active-duty US military & veterans)**										
**Kip et al. (2015)**	NA	Secondary: *civilian vs military by gender & Hx of sexual trauma*	Civilians, *n* = 62 [M_{Age} = 41, 86% female, 89% white]. Military, *n* = 51 [M_{Age} = 42, 16% female, 84% white]	30/143 (21%) 22/113 (20%) 52/143 (36%)	≥18yrs; PCL-C/M ≥ 40, or PDSQ PTSD subscale ≥ 5	1-5x (*M* = 3.8) 60-75min sessions	NA	NA	NA	SAMHSA; DoD
**Hardwick (2017)**	NA	Dissertation – pilot study: *comorbid PTSD & sleep disturbance.* Secondary: *sleep issues in civilians vs veterans*	1) Pilot study: veterans[g]: $n_{enrolled}$ = 10, $n_{completed}$ = 8 [M_{Age} = 38, 13% female, 88% white]. 2) Pooled data: Civilians & veterans, $n_{completed}$ = 125. [M_{Age} = 41, 55% female, NR% white]	Pilot Study: 0/8 (0%) Pooled Data: 12/125 (10%) 22/113 (20%) 34/125 (27%)	Pilot study: ≥18yrs; PCL-M ≥ 44; PSQI ≥ 5. Pooled data: 21-60/≥18yrs; PCL-C/PCL-M ≥ 40, or PDSQ PTSD subscale ≥ 5	1-5x (*M* = *NR*) 60-75min sessions	NA	NA	Pilot Study: NR	US Army TATRC†
**Pooled Data from Registered Clinical Trials NCT 01559688 ($n_{enrolled}$ = 57 active-duty US military & veterans) & NCT02030522($n_{enrolled}$ = 140† US service members)**										
**Kip et al. (2019)**	NA	Secondary – sub-group analyses: *1. TBI status and 2. SOF status*[h]	TBI: US service members & veterans, *n* = 202. [M_{Age} = 43, 10% female, 84% white]. SOF: US service members & veterans, *n* = 144 (*n* = 25 were SOF) [M_{Age} = 43, 0% female, 86% white]	TBI analysis: 41/202 (20%) SOF analysis: 32/141 (23%)	≥18yrs; PCL-M ≥ 40, or PDSQ PTSD subscale ≥ 5 *(TBI status: none, mild, moderate/severe)*	Modified ART	NA	NA	NA	DoD; Chris T. Sullivan Foundation

Note. In all studies ART was administered in an individual format by one or more mental health professionals formally trained in the administration of ART.

Abbreviations. ART = Accelerated Resolution Therapy; PTSD = Post-Traumatic Stress Disorder; NA = Not Applicable; NR = Not Reported; USF = University of South Florida; SAMHSA = Substance Abuse and Mental Health Services Administration; DoD = Department of Defense; RCT = Randomized Controlled Trial; Tx = Treatment; PCL = PTSD Checklist; PCL-C = PTSD Checklist Civilian; PCL-M = PTSD Checklist Military; NOSC = Naval Operational Support Center; AFB = Air Force Base; HEP = Homeless Emergency Project; Hx = History; NIH – National Institutes of Health; TATRC = Telemedicine & Advanced Technology Research Center; TBI = Traumatic Brain Injury; SOF = Special Operations Forces; ASD = Acute Stress Disorder; ICQ = Inventory of Complicated Grief.

† Information provided upon request by corresponding author.

[a] Based on the National Institutes of Health (NIH) quality rating system: good, fair, poor, cannot determine (CD), not applicable (NA), not reported (NR).
[b] Number of participants who did not complete treatment. If applicable, the second under-lined line is the number of participants lost to follow-up, and the third line is sum of treatment and follow-up attrition.
[c] $n_{enrolled}$ refers to the number of participants enrolled in an intervention, not to the number of participants screened for a study.
[d] $n_{completed}$ refers to the number of participants who completed the intervention and for which both a pre & post-intervention score was reported; however, this number does not refer to the number of participants for which 1,2, or 3-month follow-up scores were reported in applicable studies (in many cases the number of participants who completed follow-up measures is lower).
[e] Participants were stratified based on whether they had previously received no treatment for PTSD, pharmacotherapy, 1st line psychotherapy (CPT &/or PE), or another psychotherapy.
[f] Majority were civilian; however, 6 participants in the 'civilian study' were veterans with traumas specific to military service.
[g] Veterans enrolled in pilot study were drawn exclusively from participants of Registered Clinical Trial (NCT01559688).
[h] SOF sub-group analysis only used data from NCT02030522.
