# Supplementary material for: Accelerated Resolution Therapy (ART) for the treatment of posttraumatic stress disorder in adults: A systematic review
Source: PLOS Ment Health. 2024 Sep 17;1(4):e0000123. doi: 10.1371/journal.pmen.0000123 (PMC12798211; doi:10.1371/journal.pmen.0000123)
Supplement: S2 Table — (PDF) [file pmen.0000123.s008.pdf]

S2 Table. Summary statistics and key findings reported by authors of primary studies included in the systematic review.

Author (Year)	Group	Outcome Measures	Mean (Standard Deviation)$_n$ at each available time point				Key Findings Reported by Author(s)
			Pre Tx	Post Tx	2–3m FU	4–6m FU	
**Randomized Controlled Trials**							
**2011-2013 – Registered Clinical Trial (NCT01559688):** Accelerated Resolution Therapy for Psychological Trauma ($n_{enrolled}$ = 57 active-duty US military & veterans)							
Kip, Rosenzweig, et al. (2013)	ART	PCL-M (PTSD)	57.4 (15.0)$_{26}$	40.2 (13.4)$_{26}$			In this first controlled, unblinded trial of ART and with a central focus on combat-related psychological trauma, we observed clinically and statistically significant reductions in symptoms of PTSD and related comorbidities among participants assigned to the ART intervention. No appreciable changes in symptoms were observed among participants randomly assigned to the AC regimen, thereby seemingly negating the explanation of the effect of ART being attributed simply to personal interaction with a professional. Favorable treatment results for the ART group and the AC group who crossed over to ART were sustained at 3 months and are consistent with previous treatment results in civilians.
		BSI (psychological distress)	27.1 (NP)$_{26}$	12.9 (NP)$_{26}$			
		CES-D (depression)	26.7 (NP)$_{26}$	14.3 (NP)$_{26}$			
		STICSA (somatic anxiety)	18.3 (NP)$_{26}$	15.3 (NP)$_{26}$			
		STICSA (cognitive anxiety)	23.7 (NP)$_{26}$	16.5 (NP)$_{26}$			
		PSQI (sleep)	12.8 (NP)$_{26}$	10.4 (NP)$_{26}$			
	AC	PCL-M (PTSD)	56.4 (15.7)$_{24}$	53.9 (6.0)$_{24}$			
		BSI (psychological distress)	28.1 (NP)$_{24}$	24.0 (NP)$_{24}$			
		CES-D (depression)	26.9 (NP)$_{24}$	28.2 (NP)$_{24}$			
		STICSA (somatic anxiety)	20.6 (NP)$_{24}$	19.7 (NP)$_{24}$			
		STICSA (cognitive anxiety)	23.8 (NP)$_{24}$	22.3 (NP)$_{24}$			
		PSQI (sleep)	11.7 (NP)$_{24}$	11.7 (NP)$_{24}$			
	Total finished ART w/ FU data	PCL-M (PTSD)	53.4 (NP)$_{38}$		32.9 (*12.4*)$_{38}$		
		BSI (psychological distress)	24.0 (NP)$_{37}$		8.8 (NP)$_{37}$		
		CES-D (depression)	24.8 (NP)$_{38}$		13.0 (NP)$_{38}$		
		STICSA (somatic anxiety)	17.2 (NP)$_{37}$		13.6 (NP)$_{37}$		
		STICSA (cognitive anxiety)	21.2 (NP)$_{37}$		15.5 (NP)$_{37}$		
		PSQI (sleep)	11.6 (NP)$_{30}$		8.8 (NP)$_{30}$		
**2018-2019 – Registered Clinical Trial (NCT03484338):** Accelerated Resolution Therapy for Complicated Grief ($n_{enrolled}$ = 54 informal hospice caregivers)							
Buck et al. (2020)	ART	PCL-5 (PTSD)	44.1 (11.8)$_{32}$	13.2 (9.8)$_{32}$	LP (LP)$_{43}$		We observed overall better than expected results in hospice caregivers with large effect sizes across all symptoms (PTSD, and depression) irrespective of baseline symptom levels or whether ART was delivered by one or multiple interventionists.
		CES-D (depression)	29.8 (11.1)$_{30}$	14.9 (10.7)$_{30}$	LP (LP)$_{43}$		
	WL	PCL-5 (PTSD)	38.1 (15.1)$_{18}$	32.2 (13.2)$_{18}$			
		CES-D (depression)	31.1 (10.5)$_{18}$	28.4 (11.3)$_{18}$			
**Observational Trials & Studies**							
**2013-2015 – Registered Clinical Trial (NCT02030522):** Prospective Cohort Study of ART for the Treatment of Military Psychological Trauma ($n_{enrolled}$ = 140† US service members)							
Kip et al. (2016)	ART	***Homeless Vets***					Reduction of symptoms of PTSD was substantial and not significantly greater among homeless veterans vs. those treated from the community (*p* = .14), as were comorbidity reductions in depression, anxiety, and sleep quality. Results at 6-month posttreatment follow-up were similar. ART appears to be an effective, brief treatment for symptoms of PTSD among veterans residing in a homeless shelter.
		PCL-M (PTSD)	63.7 (10.3)$_{12}$	35.9 (NP)$_{12}$		36.8 (NP)$_{8}$	
		BSI (psychological distress)	27.7 (9.2)$_{12}$	11.8 (NP)$_{12}$			
		CES-D (depression)	30.4 (9.7)$_{12}$	14.8 (NP)$_{12}$			
		STICSA (state anxiety)	47.0 (11.5)$_{12}$	27.9 (NP)$_{12}$			
		PSQI (sleep)	13.9 (3.1)$_{12}$	10.1 (NP)$_{12}$			

		Housed Vets					
		PCL-M (PTSD)	62.8 (10.4)$_{77}$	41.2 (NP)$_{77}$		46.1 (NP)$_{37}$	
		BSI (psychological distress)	31.0 (12.9)$_{77}$	13.9 (NP)$_{77}$			
		CES-D (depression)	29.9 (11.0)$_{77}$	16.9 (NP)$_{77}$			
		STICSA (state anxiety)	45.9 (10.7)$_{77}$	33.9 (NP)$_{77}$			
		PSQI (sleep)	13.8 (3.9)$_{77}$	10.7 (NP)$_{77}$			
**2012 – Unregistered Study (Kip et al.):** Brief Treatment of Symptoms of Post-Traumatic Stress Disorder by Use of Accelerated Resolution Therapy ($n_{enrolled}$ = 80 mostly civilians)							
Kip et al. (2012)	ART	PCL-C (PTSD)	54.5 (12.2)$_{54}$	31.2 (11.4)$_{54}$	30.0 (12.4)$_{54}$	NP	For the PCL-C … a score of ≥44 has been shown to maximize diagnostic efficiency. Using this cutoff score, respective percentages of participants meeting this diagnostic criterion of PTSD before ART, after ART, and at 2-month follow-up were 80% versus 17% versus 15%. In this first empirical report of ART, we observed substantial reductions in self-report symptoms of PTSD, depression, anxiety, and global physical and psychological symptoms, and improvements in sleep quality after a median of 4 sessions. Favorable results were consistently observed … at 2-month post-treatment follow-up.
		BSI (psychological distress)	30.8 (14.6)$_{54}$	10.1 (10.8)$_{54}$	10.1 (12.1)$_{54}$	NP	
		CES-D (depression)	29.5 (10.9)$_{54}$	11.8 (11.1)$_{54}$	13.5 (12.1)$_{54}$	NP	
		STICSA (somatic anxiety)	20.6 (6.9)$_{54}$	13.8 (3.5)$_{54}$	14.9 (5.0)$_{54}$	NP	
		STICSA (cognitive anxiety)	25.2 (6.6)$_{54}$	15.5 (5.0)$_{54}$	17.5 (6.8)$_{54}$	NP	
		PSQI (sleep)	9.2 (4.5)$_{46}$	6.4 (4.4)$_{46}$	7.0 (4.6)$_{45}$	NP	
**2017 – Unregistered Pilot Study (Rossiter et al.):** Accelerated Resolution Therapy for women veterans experiencing military sexual trauma related post-traumatic stress disorder							
Rossiter et al. (2017)	ART	PCL-M (PTSD)	62.2 (*8.7*)$_{5}$	33.8 (*8.7*)$_{5}$			After delivery of ART, both statistically and clinically significant improvements were reported for symptoms of military sexual assault-related PTSD (MST-PTSD) including psychological trauma, psychological distress, sleep, depression, and anxiety. Results from this feasibility study indicate that ART is a viable treatment option for women with MTS-PTSD.
		BSI (psychological distress)	29.2 (*11.7*)$_{5}$	8.0 (*11.7*)$_{5}$			
		CES-D (depression)	22.0 (*8.1*)$_{5}$	16.6 (*8.1*)$_{5}$			
		STICSA (state anxiety)	40.0 (*11.6*)$_{5}$	27.2 (*11.6*)$_{5}$			
		PSQI (sleep)	20.0 (*8.9*)$_{5}$	15.6 (*8.9*)$_{5}$			

Note. Empty cells indicate no data collected for that time point. Italicized summary statistics indicate values calculated by the reviewer using data available within the study (e.g., obtaining standard deviations from *p*-values, *t*-statistics, &/or standard errors) or imputed based on value obtained from another study with comparable sample at the relevant time-point. All non-italicized summary statistics are as reported in each study.

Abbreviations. NP = Not Provided in publication & not available upon request; LP = Low Precision (e.g., boxplot provided, but no precise values); ART = Accelerated Resolution Therapy; PTSD = Post-Traumatic Stress Disorder; FU = Follow-up; PCL = PTSD Checklist; PCL-C = PTSD Checklist Civilian; PCL-M = PTSD Checklist Military; BSI = Brief Symptom Inventory; CES-D = Center for Epidemiological Studies Depression Scale; STICSA = State-Trait Inventory for Cognitive and Somatic Anxiety; PSQI = Pittsburgh Sleep Quality Index; AC = Attention Control; Vets = Veterans; CST = Civilian Sexual Trauma; MST = Military Sexual Trauma; NR = Not Reported; TBI = Traumatic Brain Injury; SOF = Special Operations Forces; ASD = Acute Stress Disorder; Dx = Diagnosis.

† Information provided upon request by corresponding author.
