# Supplementary material for: Accelerated Resolution Therapy (ART) for the treatment of posttraumatic stress disorder in adults: A systematic review
Source: PLOS Ment Health. 2024 Sep 17;1(4):e0000123. doi: 10.1371/journal.pmen.0000123 (PMC12798211; doi:10.1371/journal.pmen.0000123)
Supplement: S3 Table — (PDF) [file pmen.0000123.s009.pdf]

S3 Table. Summary statistics and key findings reported by authors of reports of studies included in the systematic review.

Author (Year)	Group	Outcome Measures	Mean (Standard Deviation)$_n$ at each available time point				Key Findings Reported by Author(s)
			Pre Tx	Post Tx	2–3m FU	4–6m FU	
			**Observational Trials & Studies**				
**2013-2015 – Registered Clinical Trial (NCT02030522): Prospective Cohort Study of Accelerated Resolution Therapy for the Treatment of Military Psychological Trauma ($n_{enrolled}$ = 140[†] US service members)**							
**Witt (2019)[a]**	ART	***Low PTSD Severity*** *(40-50)*					The number of deployments, depression, & anxiety were nonsignificant predictors of PTSD symptom reduction in this study. ART resulted in clinically meaningful mean PTSD symptom reduction of 10 or more points as measured by the PCL-M scale scores of all three PTSD symptom severity groups. Reduction in PTSD symptoms was significantly greater in the high severity group as compared to the low severity group.
		PCL-M (PTSD)	NP (*9.3*)$_{27}$	-14.5 (*9.3*)$_{27}$			
		Moderate Severity (51-60)					
		PCL-M (PTSD)	NP (*11.6*)$_{26}$	-19.5(*11.6*)$_{26}$			
		High PTSD Severity (≥61)					
		PCL-M (PTSD)	NP (*18.4*)$_{55}$	-26.1(*18.4*)$_{55}$			
**Pang et al. (2021)**	ART	***Treatment Naive***					Among treatment completers, the clinically significant treatment response rate (reduction of ≥10 points on the PCL-M) were highest in the treatment-naïve (83%) and first-line psychotherapy (88%) groups. Similar significant symptom reductions were observed for measures of depression and anxiety, and favorable treatment effects were generally sustained at 6-month follow-up.
		PCL-M (PTSD)	52.3 (15.5)$_{33}$	27.2 (NP)$_{18}$		24.0 (NP)$_{8}$	
		BSI (psychological distress)	24.5 (15.3)$_{33}$	7.4 (NP)$_{18}$		0.1 (NP)$_{8}$	
		CES-D (depression)	24.2 (12.2)$_{33}$	9.6 (NP)$_{18}$		6.3 (NP)$_{8}$	
		STICSA (somatic anxiety)	20.2 (6.7)$_{33}$	13.9 (NP)$_{18}$		13.6 (NP)$_{8}$	
		STICSA (cognitive anxiety)	22.8 (8.1)$_{33}$	13.0 (NP)$_{18}$		10.8 (NP)$_{8}$	
		PSQI (sleep)	12.0 (3.8)$_{33}$	7.1 (NP)$_{18}$		6.7 (NP)$_{8}$	
		Pharmacotherapy Only					In a brief treatment period, ART appears to result in substantial reductions in symptoms of PTSD among veterans, including those with residual PTSD symptoms after prior treatment with first-line psychotherapies endorsed by the U.S. Department of Defense and Veterans Affairs. These results suggest that ART be considered as a potential first-line treatment modality for veterans with PTSD.
		PCL-M (PTSD)	58.8 (12.2)$_{40}$	43.4 (NP)$_{31}$		48.9 (NP)$_{15}$	
		BSI (psychological distress)	28.6 (12.4)$_{40}$	14.6 (NP)$_{31}$		19.0 (NP)$_{15}$	
		CES-D (depression)	30.2 (11.0)$_{40}$	18.6 (NP)$_{31}$		21.3 (NP)$_{15}$	
		STICSA (somatic anxiety)	20.7 (6.3)$_{40}$	17.3 (NP)$_{31}$		21.1 (NP)$_{15}$	
		STICSA (cognitive anxiety)	25.3 (7.3)$_{40}$	18.2 (NP)$_{31}$		22.6 (NP)$_{15}$	
		PSQI (sleep)	13.9 (3.8)$_{40}$	12.2 (NP)$_{31}$		12.3 (NP)$_{15}$	
		1st Line Psychotherapy					
		PCL-M (PTSD)	64.6 (9.9)$_{33}$	40.5 (NP)$_{25}$		43.2 (NP)$_{16}$	
		BSI (psychological distress)	32.3 (14.1)$_{33}$	14.7 (NP)$_{25}$		18.5 (NP)$_{16}$	
		CES-D (depression)	29.6 (10.9)$_{33}$	18.1 (NP)$_{25}$		21.4 (NP)$_{16}$	
		STICSA (somatic anxiety)	20.5 (6.1)$_{33}$	15.3 (NP)$_{25}$		17.9 (NP)$_{16}$	
		STICSA (cognitive anxiety)	25.9 (6.7)$_{33}$	15.7 (NP)$_{25}$		19.7 (NP)$_{16}$	
		PSQI (sleep)	15.0 (3.3)$_{33}$	11.0 (NP)$_{25}$		12.2 (NP)$_{16}$	
		Other Psychotherapy					
		PCL-M (PTSD)	58.4 (14.8)$_{42}$	39.2 (NP)$_{32}$		47.6 (NP)$_{16}$	
		BSI (psychological distress)	27.1 (13.6)$_{42}$	12.5 (NP)$_{32}$		20.7 (NP)$_{16}$	
		CES-D (depression)	25.9 (11.3)$_{42}$	13.2 (NP)$_{32}$		20.9 (NP)$_{16}$	
		STICSA (somatic anxiety)	19.7 (4.8)$_{42}$	15.5 (NP)$_{32}$		19.2 (NP)$_{16}$	

		STICSA (cognitive anxiety)	22.9 (6.8)$_{42}$	16.2 (NP)$_{32}$		20.9 (NP)$_{16}$	
		PSQI (sleep)	12.6 (4.0)$_{42}$	10.1 (NP)$_{32}$		12.4 (NP)$_{16}$	
**2012 – Unregistered Study (Kip et al.): Brief Treatment of Symptoms of Post-Traumatic Stress Disorder by Use of Accelerated Resolution Therapy ($n_{enrolled}$ = 80 mostly civilians)**							
**Kip, Sullivan, et al. (2013)**	ART	PCL-C (PTSD)	62.5 (8.8)$_{28}$	32.9 (12.5)$_{28}$	32.4 (13.1)$_{28}$	31.1 (10.4)$_{19}$	With respect to treatment with ART, reductions in depressive symptoms appear to occur in close concordance with reductions in all symptoms of PTSD. While there appeared to be a slight attenuation of treatment effect over time for depression (CES-D scores), effect sizes were still large and suggestive of sustained treatment response for symptoms of both PTSD and depression. In summary, ART appears to be a promising brief, safe, and effective treatment for adults with clinically significant comorbid symptoms of PTSD & depression.
		BSI (psychological distress)	39.2 (13.7)$_{28}$	13.7 (11.9)$_{28}$	13.6 (13.9)$_{28}$	14.5 (14.1)$_{19}$	
		CES-D (depression)	35.1 (8.8)$_{28}$	14.5 (11.0)$_{28}$	17.0 (11.5)$_{28}$	19.5 (14.4)$_{19}$	
		STICSA (somatic anxiety)	24.0 (7.1)$_{28}$	15.1 (6.8)$_{28}$	16.5 (5.9)$_{28}$	17.7 (5.4)$_{19}$	
		STICSA (cognitive anxiety)	28.8 (5.1)$_{28}$	16.5 (5.3)$_{28}$	19.9 (7.2)$_{28}$	19.7 (7.2)$_{19}$	
		PSQI (sleep)	11.4 (4.0)$_{28}$	8.0 (3.5)$_{28}$	8.2 (4.0)$_{28}$	7.0 (4.2)$_{19}$	
**Pooled Data from Kip et al., 2012 ($n_{enrolled}$ = 80 mostly civilians) and Registered Clinical Trial NCT01559688 ($n_{enrolled}$ = 57 active-duty US military & veterans)**							
**Kip et al. (2015)**	ART	***Civilian***					There was no significant differential response to treatment based on military vs civilian status after adjusting for history of head trauma and sleep quality [*effect sizes in maroon had to be calculated using pre-adjusted values*]; however, females with MST had a less profound response to treatment than those with CST. 10 of 14 females (71.4%) who completed treatment with ART for CST had a reduction of ≥10 points on the PCL, a cut point used to define statistical and clinically meaningful change (improvement). Similarly, 4 of the 6 females (66.7%) who completed treatment with ART for MST had a reduction of ≥10 points on the PCL.
		PCL-C (PTSD)	53.2 (12.2)$_{62}$	30.2(11.2)$_{62}$	NP (NP)$_{50}$		
		Intrusion subscale	16.1 (4.3)$_{62}$	8.6 (3.0)$_{62}$	NP		
		Arousal subscale	14.5 (5.2)$_{62}$	NP	NP		
		Avoidance subscale	6.5 (2.2)$_{62}$	NP	NP		
		Numbing subscale	16.0 (4.7)$_{62}$	9.4 (4.1)$_{62}$	NP		
		BSI (psychological distress)	30.0 (14.7)$_{62}$	NP	NP		
		CES-D (depression)	27.8 (11.8)$_{62}$	NP	NP		
		PSQI (sleep)	9.0 (4.3)$_{62}$	NP	NP		
		Military					
		PCL-M (PTSD)	56.0 (14.8)$_{51}$	40.5 (17.2)$_{51}$	NP (NP)$_{41}$		
		Intrusion subscale	16.1 (5.1)$_{51}$	11.6 (5.5)$_{51}$	NP		
		Arousal subscale	18.0 (4.5)$_{51}$	NP	NP		
		Avoidance subscale	6.8 (2.5)$_{51}$	NP	NP		
		Numbing subscale	15.1 (5.4)$_{51}$	11.3 (5.6)$_{51}$	NP		
		BSI (psychological distress)	25.6 (16.9)$_{51}$	NP	NP		
		CES-D (depression)	26.8 (14.0)$_{51}$	NP	NP		
		PSQI (sleep)	12.3 (4.2)$_{51}$	NP	NP		
		Female CST					
		PCL-C (PTSD)	52.9 (12.1)$_{14}$	30.4 (12.7)$_{14}$	24.5 (8.9)$_{11}$		
		Female MST					
		PCL-M (PTSD)	68.7 (8.2)$_{6}$	47.5 (16.3)$_{6}$	44.8 (15.2)$_{5}$		
**Hardwick (2017)**	ART	***Pilot Study***					1) Pilot study: there was confirmatory evidence that ART resulted in improved subjective reported sleep quality.
		PCL-M (PTSD)	63.8 (10.4)$_{8}$	33.6 (13.3)$_{8}$			
		PSQI (sleep)	14.5 (4.4)$_{8}$	9.6 (5.4)$_{8}$			
		Civilian (pooled)					2) Pooled data: there was no significant differential effect of ART on sleep disturbance in civilian vs veteran samples, particularly at 3-month follow-up.
		PCL-C (PTSD)	54.2 (12.7)$_{75}$	30.7(11.2)$_{NP}$	30.0(12.4)$_{NP}$		

		PSQI (sleep)	9.4 (4.4)$_{NP}$	6.7 (4.6)$_{NP}$	6.7 (4.5)$_{NP}$	
		Military (pooled)				
		PCL-M (PTSD)	56.1 (15.3)$_{50}$	40.7(17.8)$_{NP}$	33.1(12.7)$_{NP}$	
		PSQI (sleep)	12.6 (4.3)$_{NP}$	33.1(12.7)$_{NP}$	8.8 (5.5)$_{NP}$	
**Pooled Data from Registered Clinical Trials NCT 01559688 ($n_{enrolled}$ = 57 active-duty US military & veterans) & NCT02030522($n_{enrolled}$ = 140† US service members)**						
**Kip et al. (2019)**	ART	***No TBI***				After statistical adjustment for potential confounding variables, the three groups did not differ statistically in the between-group mean reduction in PTSD symptoms (p = 0.15). Using the definition of clinically meaningful change (reduction) in symptoms of PTSD (≥10-point reduction), respective percentages were 69.3% in the no TBI group, 76.3% in the mild TBI group and 65.7% in the moderate/severe TBI group (p = 0.59). Imputing all treatment non-completers as having no treatment response resulted in corresponding treatment response rates of 58.1%, 60.4% and 46.9%, p = 0.33. In examining change in comorbidities associated with PTSD, the three groups experienced similar medium-to-large reductions (effect sizes) on the Brief Symptom Inventory, depression, and sleep function. However, the moderate/severe TBI group experienced less reduction in anxiety compared to the no and mild TBI groups.
		PCL-M (PTSD)	56.7(14.0)$_{105}$	37.9 (NP)$_{88}$		
		BSI (psychological distress)	26.7(14.5)$_{105}$	11.8 (NP)$_{88}$		
		CES-D (depression)	28.1(12.5)$_{105}$	15.2 (NP)$_{88}$		
		STICSA (state anxiety)	43.1(12.4)$_{105}$	30.6 (NP)$_{88}$		
		PSQI (sleep quality)	12.4 (4.1)$_{105}$	9.9 (NP)$_{88}$		
		Mild TBI				
		PCL-M (PTSD)	58.4 (14.9)$_{48}$	37.2 (NP)$_{38}$		
		BSI (psychological distress)	26.4 (15.3)$_{48}$	10.3 (NP)$_{38}$		
		CES-D (depression)	26.2 (12.7)$_{48}$	11.9 (NP)$_{38}$		
		STICSA (state anxiety)	44.2 (13.3)$_{48}$	30.1 (NP)$_{38}$		
		PSQI (sleep quality)	12.8 (4.2)$_{48}$	9.8 (NP)$_{38}$		
		Moderate TBI				
		PCL-M (PTSD)	62.3 (11.2)$_{49}$	44.8 (NP)$_{35}$		
		BSI (psychological distress)	31.2 (14.9)$_{49}$	17.5 (NP)$_{35}$		Using the definition of clinically meaningful change (reduction) in symptoms of PTSD (≥10-point reduction), respective percentages were 75.0% in the non-SOF group vs. 60.0% in the SOF group (p = 0.14). Imputing the treatment non-completers as having no treatment response resulted in corresponding treatment response rates of 54.3% vs. 60.0%, respectively (p = 0.60). In examining change in comorbidities associated with PTSD, both groups experienced clinically meaningful changes (improvement), yet the non-SOF group appeared to have better treatment response than the SOF group for symptoms of depression and sleep quality.
		CES-D (depression)	28.0 (12.1)$_{49}$	18.9 (NP)$_{35}$		
		STICSA (state anxiety)	45.0 (11.3)$_{49}$	37.3 (NP)$_{35}$		
		PSQI (sleep quality)	14.5 (3.6)$_{49}$	11.0 (NP)$_{35}$		
		Non-SOF				
		PCL-M (PTSD)	59.6(12.3)$_{116}$	37.6 (NP)$_{84}$		
		BSI (psychological distress)	27.9(13.7)$_{116}$	11.4 (NP)$_{84}$		
		CES-D (depression)	27.1(10.9)$_{116}$	14.4 (NP)$_{84}$		
		STICSA (state anxiety)	44.4(11.3)$_{116}$	31.7 (NP)$_{84}$		
		PSQI (sleep quality)	13.0(4.0)$_{116}$	9.9 (NP)$_{84}$		
		SOF				
		PCL-M (PTSD)	58.6(16.2)$_{25}$	44.1 (NP)$_{25}$		
		BSI (psychological distress)	28.6(16.2)$_{25}$	16.0 (NP)$_{25}$		
		CES-D (depression)	28.4(14.5)$_{25}$	18.0 (NP)$_{25}$		
		STICSA (state anxiety)	42.8(13.5)$_{25}$	32.8 (NP)$_{25}$		
		PSQI (sleep quality)	14.2(4.2)$_{25}$	11.9 (NP)$_{25}$		

Note. Empty cells indicate no data collected for that time point. Italicized summary statistics indicate values calculated by the reviewer using data available within the study (e.g., obtaining standard deviations from *p*-values, *t*-statistics, &/or standard errors) or imputed based on value obtained from another study with comparable sample at the relevant time-point. All non-italicized summary statistics are as reported in each study.

Abbreviations. NP = Not Provided in publication & not available upon request; LP = Low Precision (e.g., boxplot provided, but no precise values); ART = Accelerated Resolution Therapy; PTSD = Post-Traumatic Stress Disorder; FU = Follow-up; PCL = PTSD Checklist; PCL-C = PTSD Checklist Civilian; PCL-M = PTSD Checklist Military; BSI = Brief Symptom Inventory; CES-D = Center for Epidemiological Studies Depression Scale; STICSA = State-Trait Inventory for Cognitive and Somatic Anxiety; PSQI = Pittsburgh Sleep Quality Index; AC = Attention Control; Vets = Veterans; CST = Civilian Sexual Trauma; MST = Military Sexual Trauma; NR = Not Reported; TBI = Traumatic Brain Injury; SOF = Special Operations Forces; ASD = Acute Stress Disorder; Dx = Diagnosis.

† Information provided upon request by corresponding author.

[a] Did not report pre and post means, only a change score.
