# Supplementary material for: Accelerated Resolution Therapy (ART) for the treatment of posttraumatic stress disorder in adults: A systematic review
Source: PLOS Ment Health. 2024 Sep 17;1(4):e0000123. doi: 10.1371/journal.pmen.0000123 (PMC12798211; doi:10.1371/journal.pmen.0000123)
Supplement: S4 Table — (PDF) [file pmen.0000123.s010.pdf]

S4 Table. Outcomes of reports of studies included in the systematic review.

Author (Year)	Analyses	Follow-up	Group	Outcome Measures	Within group effect size (*d*)[a] [95% CI] for ART			Between groups effect size (*d*)[a] at each available time point			
					Pre to Post	Pre to 2–3m FU	Pre to 4–6m FU	Pre Tx	Post Tx	2–3m FU	4–6m FU
**2013-2015 – Registered Clinical Trial (NCT02030522): Prospective Cohort Study of Accelerated Resolution Therapy for the Treatment of Military Psychological Trauma ($n_{enrolled}$ = 140† US service members)**											
**Witt (2019)**	Completer	Post	ART	***Low PTSD Severity*** *(40-50)*				***Low vs High***			
				PCL-M (PTSD)	NP			NP	0.38 [NP]		
				Moderate Severity (51-60)							
				PCL-M (PTSD)	NP						
				High PTSD Severity (≥61)							
				PCL-M (PTSD)	NP						
**Pang et al. (2021)**	Completer	Post<br>6 months	ART	***Treatment Naive***							
				PCL-M (PTSD)	1.48 [NP]		1.48 [NP]				
				BSI (psych distress)	1.16 [NP]		2.11 [NP]				
				CES-D (depression)	1.02 [NP]		1.32 [NP]				
				STICSA (somatic anx)	1.18 [NP]		1.38 [NP]				
				STICSA (cognitive anx)	0.98 [NP]		0.95 [NP]				
				PSQI (sleep)	1.25 [NP]		0.89 [NP]				
				Pharmacotherapy Only							
				PCL-M (PTSD)	1.11 [NP]		0.80 [NP]				
				BSI (psych distress)	1.09 [NP]		0.64 [NP]				
				CES-D (depression)	1.00 [NP]		0.57 [NP]				
				STICSA (somatic anx)	1.19 [NP]		0.31 [NP]				
				STICSA (cognitive anx)	0.60 [NP]		0.05 [NP]				
				PSQI (sleep)	0.51 [NP]		0.36 [NP]				
				1st Line Psychotherapy							
				PCL-M (PTSD)	1.88 [NP]		1.53 [NP]				
				BSI (psych distress)	1.57 [NP]		0.91 [NP]				
				CES-D (depression)	1.06 [NP]		0.86 [NP]				
				STICSA (somatic anx)	1.43 [NP]		0.84 [NP]				
				STICSA (cognitive anx)	0.85 [NP]		0.35 [NP]				
				PSQI (sleep)	0.78 [NP]		0.54 [NP]				
				Other Psychotherapy							
				PCL-M (PTSD)	1.03 [NP]		0.53 [NP]				
				BSI (psych distress)	1.06 [NP]		0.36 [NP]				
				CES-D (depression)	1.12 [NP]		0.36 [NP]				
				STICSA (somatic anx)	0.81 [NP]		0.24 [NP]				
				STICSA (cognitive anx)	0.65 [NP]		0.09 [NP]				
				PSQI (sleep)	0.61 [NP]		0.07 [NP]				

**2012 – Unregistered Study (Kip et al.): Brief Treatment of Symptoms of Post-Traumatic Stress Disorder by Use of Accelerated Resolution Therapy ($n_{enrolled}$ = 80 mostly civilians)**										
**Kip, Sullivan, et al. (2013)**	Completer	Post	ART	PCL-C (PTSD)	2.37 [1.61,3.13]	2.30 [1.57,3.03]	3.01 [1.94,4.08]			
		2 months		BSI (psych distress)	2.14 [1.45,2.83]	1.85 [1.23,2.46]	1.75 [0.98,2.52]			
		4 month[b]		CES-D (depression)	1.88 [1.25,2.52]	1.58 [1.04,2.12]	1.09 [0.44,1.74]			
				STICSA (somatic anxiety)	1.30 [0.76,1.85]	1.27 [0.79,1.74]	1.16 [0.62,1.71]			
				STICSA (cognitive anxiety)	2.34 [1.61,3.07]	1.24 [0.72,1.76]	1.28 [0.68,1.88]			
				PSQI (sleep)	0.97 [0.57,1.37]	0.80 [0.40,1.20]	1.05 [0.36,1.74]			
**Pooled Data from Kip et al., 2012 ($n_{enrolled}$ = 80 mostly civilians) and Registered Clinical Trial NCT01559688 ($n_{enrolled}$ = 57 active-duty US military & veterans)**										
**Kip et al. (2015)**	Completer	Post	ART	***Civilian***				***Civ vs Mil***		
				PCL-C (PTSD)	NP	NP		NP	NP	NP
		2 or 3 months		Intrusion subscale	NP	NP				
				Arousal subscale	NP	NP				
				Avoidance subscale	NP	NP				
				Numbing subscale	NP	NP				
				BSI (psych distress)	NP	NP				
				CES-D (depression)	NP	NP				
				PSQI (sleep)	NP	NP				
				Military						
				PCL-M (PTSD)	NP	NP				
				Intrusion subscale	NP	NP				
				Arousal subscale	NP	NP				
				Avoidance subscale	NP	NP				
				Numbing subscale	NP	NP				
				BSI (psych distress)	NP	NP				
				CES-D (depression)	NP	NP				
				PSQI (sleep)	NP	NP				
				Female CST				***CST vs MST***		
				PCL-C (PTSD)	NP	NP		NP	NP	NP
				Female MST						
				PCL-M (PTSD)	NP	NP				
**Hardwick (2017)**	Completer	Post	ART	***Pilot Study***						
		3 months (pooled only)		PCL-M (PTSD)	2.53 [NP]					
				PSQI (sleep)	1.00 [NP]					
				Civilian (pooled)				***Civ vs Mil***		
				PCL-C (PTSD)				NP	1.77 [NP]	0.29 [NP]
				PSQI (sleep)				NP	0.00 [NP]	−0.44 [NP]
				Military (pooled)						
				PCL-M (PTSD)						
				PSQI (sleep)						

**Pooled Data from Registered Clinical Trials NCT 01559688 ($n_{enrolled}$ = 57 active-duty US military & veterans) & NCT02030522($n_{enrolled}$ = 140[†] US service members)**					
**Kip et al. (2019)**	Completer	Post	ART	***No TBI***	
				PCL-M (PTSD)	1.14 [NP]
				BSI (psych distress)	1.12 [NP]
				CES-D (depression)	1.14 [NP]
				STICSA (state anxiety)	1.02 [NP]
				PSQI (sleep quality)	0.66 [NP]
				Mild TBI	
				PCL-M (PTSD)	1.40 [NP]
				BSI (psych distress)	1.19 [NP]
				CES-D (depression)	1.20 [NP]
				STICSA (state anxiety)	1.24 [NP]
				PSQI (sleep quality)	0.80 [NP]
				Moderate TBI	
				PCL-M (PTSD)	1.25 [NP]
				BSI (psych distress)	1.00 [NP]
				CES-D (depression)	0.78 [NP]
				STICSA (state anxiety)	0.65 [NP]
				PSQI (sleep quality)	0.64 [NP]
				Non-SOF	
				PCL-M (PTSD)	1.39 [NP]
				BSI (psych distress)	1.27 [NP]
				CES-D (depression)	1.22 [NP]
				STICSA (state anxiety)	1.05 [NP]
				PSQI (sleep quality)	0.82 [NP]
				SOF	
				PCL-M (PTSD)	0.85 [NP]
				BSI (psych distress)	0.90 [NP]
				CES-D (depression)	0.73 [NP]
				STICSA (state anxiety)	0.82 [NP]
				PSQI (sleep quality)	0.48 [NP]

Note. Empty cells indicate no data collected for that time point. Within and between effect sizes and 95% CI's are as reported in each study. Positive effect sizes reflect an improvement in symptoms relative to baseline or control (or in the cases of Witt, 2019 – high PTSD symptom severity relative to low PTSD symptom severity groups; Kip et al., 2015 – civilians relative to military and civilian females with CST relative to military females with MST; and Hardwick, 2017 – civilians relative to military); negative effect sizes reflect a degradation in symptoms relative to baseline or control.

Abbreviations. NP = Not Provided in publication & not available upon request; LP = Low Precision (e.g., boxplot provided, but no precise values); ART = Accelerated Resolution Therapy; PTSD = Post-Traumatic Stress Disorder; FU = Follow-up; ITT = Intention To Treat; PCL = PTSD Checklist; BSI = Brief Symptom Inventory; CES-D = Center for Epidemiological Studies Depression Scale;

STICSA = State-Trait Inventory for Cognitive and Somatic Anxiety; PSQI = Pittsburgh Sleep Quality Index; AC = Attention Control; Vets = Veterans; CST = Civilian Sexual Trauma; MST = Military Sexual Trauma; TBI = Traumatic Brain Injury; SOF = Special Operations Forces; ASD = Acute Stress Disorder; Dx = Diagnosis.

† Information provided upon request by corresponding author.

[a] According to Cohen (1988), $d < 0.2$ indicates no difference, $d = 0.2$-0.49 indicates a small difference, $d = 0.5$-0.79 indicates a medium difference, and $d \geq 0.8$ indicates a large difference.
[b] Parent study collected but did not report 4 month follow up data.[†]
