# Supplementary material for: Accelerated Resolution Therapy (ART) for the treatment of posttraumatic stress disorder in adults: A systematic review
Source: PLOS Ment Health. 2024 Sep 17;1(4):e0000123. doi: 10.1371/journal.pmen.0000123 (PMC12798211; doi:10.1371/journal.pmen.0000123)
Supplement: Attachment — Submitted filename: Response to Reviewers.docx [file pmen.0000123.s011.docx]

I would like to thank both reviewers for taking the time out of their busy schedules to review this manuscript. It is very hard to find scholars willing and able to review nowadays so your support and thoughtful recommendations are greatly appreciated! Thankyou for helping me improve my systematic review.

NOTE: REVIEWER COMMENTS ARE IN BLACK INK AND MY RESPONSES IN BLUE INK; QUOTED MATERIALS ARE IN PURPLE INK.

**Reviewer #1:**

1) ***Intro: Although stats can vary, what is the prevalence among the gen pop, civilians?***

In addition to the prevalence estimates provided in the intro globally, I have added stats specific to the U.S. general population: “[…] According to the U.S. Department of Veterans Affairs National Center for PTSD (3) about 6% of the U.S. general population will have PTSD at *some* point in their lives and in any given year about 5% of the U.S. general population has active PTSD symptoms. In the general population PTSD is more common in women (8%) than men (4%), largely due to women’s increased exposure to sexual violence.(3) In military and veteran populations rates of PTSD are higher for both men and women. […]”

2) ***Provide brief description of the common forms of therapy to differentiate between those & ART.***

I have added in brief descriptions of the other common treatment for PTSD in the second paragraph of the introduction: “There are three interventions strongly recommended for the treatment of PTSD: trauma focused cognitive behavioural therapy (CBT), cognitive processing therapy (CPT), and prolonged exposure (PE).(7) Trauma focused CBT is a 12–16 session present focused treatment that aims to understand how a client’s current thoughts, feelings, and behaviors around a trauma are maintaining their difficulties, and then works with the client to help them get ‘unstuck’ by applying principles from both cognitive and behavioral psychology (e.g., challenging unhelpful beliefs, activity scheduling, etc.). CPT for PTSD is a 12-session treatment which focuses initially on why a trauma occurred and then emphasizes identifying, challenging, and then modifying unhelpful beliefs related to the trauma through the use of progressive worksheets. PE for PTSD is an 8–15 session treatment consisting of imaginal exposure—recounting the traumatic memory and processing the experience—and in vivo exposure—repeatedly confronting trauma-related stimuli that are now safe but were previously avoided. Additional interventions that have some support are eye movement desensitization and reprocessing (EMDR) therapy and pharmacotherapy, such as sertraline, paroxetine, fluoxetine, and venlafaxine.(8) EMDR is a 6–12 session exposure-based therapy that pairs rapid eye movements—which proponents claim facilitate processing and integration—with cognitive processing of traumatic memories.”

3) ***Can you provide sample search terms?***

This information can be found in supplementary material (S2 Appendix. Search Terms). I’m not sure if you have access to the supplementary material when reviewing. I have included a screen clipping of the appendix for your convenience:

4) ***What was your PICO statement?***

Under the ‘Eligibility Criteria’ section the PICOS statement sections were underlined. To make this clearer to the reader I have bolded the key word and added the following sentence at the beginning of this section of the manuscript to clarify this: The PICO model—Population, Intervention, Comparison, Outcome—was used to structure the eligibility criteria for this review, with the addition of ‘study design’ and ‘length of follow-up’.

Below is an excerpt from the manuscript providing the PICOS statement, with the changes I have made based on your suggestion:

The **population** of interest was adults 18 years of age or older—military or civilian—who met a clinical cut-off score for PTSD on a validated screening measure (e.g., PCL-5). The psychological **intervention** of interest was ART delivered in any setting (e.g., private practice, hospital, residential treatment centre, military deployment, etc.) by a qualified mental health professional with **comparison** to an active (e.g., CBT, pharmacotherapy, treatment as usual), or inactive control (e.g., wait-list) group. Intervention studies that did not include a control group (e.g., cohort studies, case series, feasibility studies) were also eligible. The primary **outcome** of interest was change in PTSD symptom severity from baseline to post intervention as measured by a validated scale of PTSD symptomatology. Secondary outcomes of interest included change in symptoms of psychological distress, depression, anxiety, and sleep quality. Additional outcomes of interest included treatment engagement and retention as measured by the number of sessions attended and the participant attrition rate prior to the end of treatment and prior to the last follow-up. Reports of harms possibly or probably associated with the intervention were also of interest. A wide range of **study designs** were considered including randomized controlled trials (RCTs), non-randomized trials, cohort studies, case-control studies, case series, and feasibility studies. Studies were excluded if they: a) did not utilize a quantitative design; b) were not primary studies or did not report on primary studies; c) were not peer-reviewed; and d) were in a language other than English with no English translation available. No restrictions were placed on **length of follow-up**.

5) ***Elaborate on forest plot and why this is used.***

We felt it was important to include a visual display of the result of the synthesis without meta-analysis to aid in interpretation. The forest plot without summary diamond provides a quick indicator of the effect size for each study along with a visual indicator of the relative study size, and an indication of the quality. We can see that on the whole the randomized-controlled studies provide greater precision (i.e., the confidence bands are tighter) compared to the observational studies, as would be expected, and that within each study type the higher quality studies found more modest effect sizes than the poorer studies. However, all the studies found large effect sizes. A picture is worth a thousand words.

As per the Cochrane Handbook for Systematic Reviews of Interventions ([Cochrane Handbook for Systematic Reviews of Interventions | Cochrane Training](https://training.cochrane.org/handbook/current)), Part 2, Chapter 12: Synthesizing and presenting findings using other methods: “Visual display and presentation of data is especially important for transparent reporting in reviews without meta-analysis, and should be considered irrespective of whether synthesis is undertaken […] Some mention is warranted here of their [i.e., forest plots] importance for displaying study results when meta-analysis is not undertaken (i.e. without the summary diamond). Forest plots can aid interpretation of individual study results and convey overall patterns in the data, especially when studies are ordered by a characteristic important for interpreting results (e.g. dose and effect size, sample size) […] These approaches to ordering provide important techniques for informally exploring heterogeneity in reviews without meta-analysis, and should be considered in preference to alphabetical ordering by study ID alone (Schriger et al 2010).”

Below is a pasted copy of the Fig2.tif file with the figure caption and legend included below it for reference:

**Fig 2. Forest plot of effect of ART on PTSD severity from pre- to post-intervention.**

*Note.* Quality rating is as assessed by the NIH Study Quality Assessment Tools (National Institutes of Health, 2021). The size of the point estimate boxes is scaled to log10 (N), where N = number of participants in each study who completed treatment (i.e., a larger box indicates a larger sample). The Standardized Mean Difference (SMD) is a measure of effect size and was calculated by the review authors using summary data (and therefore may differ slightly from those reported by study authors as found in Table 4). The SMDs were calculated assuming a medium association of r = .30 between pre and post intervention. Sensitivity tests were conducted with low and high associations of r = .10 and r = .50, respectively, and did not differ significantly. According to Cohen (1992), a small effect size is considered 0.2–0.49, a moderate effect size is 0.5–0.79, and a large effect size is 0.80 or greater. Note that a given effect size is not on its own indicative of whether a given effect is or is not clinically significant.

6) ***Not sure why the PCL-5 score of 33 is less stringent when 33 is the typical cutoff.***

I believe you are referring to the following paragraph: An eligibility criterion in four of the studies required participants to have a PCL-C or PCL-M score of > 40, or a PDSQ PTSD subscale score of ≥ 5 (22, 24-26). Buck, Cairns (23) required an Inventory of Complicated Grief (ICQ) score > 25 and was less stringent in that the PCL-5 score only needed to be > 33. I can see how this wording is confusing. I am stating that the Buck et al., (2020) study uses a less stringent cut-off than the other four studies (i.e., 33 vs 40). I have reworded the paragraph to read as follows: “An eligibility criterion in four of the studies required participants to have a PCL-C or PCL-M score of > 40, or a PDSQ PTSD subscale score of ≥ 5 (22, 24-26). Buck, Cairns (23) required an Inventory of Complicated Grief (ICQ) score > 25 and a PCL-5 score > 33, rather than > 40. It should be noted that a cut-point score of 31–33 on the PCL-5 is frequently used in clinical practice, but several of the studies opted to use a higher cut-point to decrease the possible number of false positives.”

From the ‘Using the PTSD checklist for DSM-5 (PCL-5)’ document published by the US Veteran’s Affairs National Center for PTSD, it states: “using a cut-point score of 31-33 appears to be reasonable based upon current psychometric work. However, when choosing a cutoff score, it is essential to consider the goals of the assessment and the population being assessed. The lower the cutoff score, the more lenient the criteria for inclusion, increasing the possible number of false-positives. The higher the cutoff score, the more stringent the inclusion criteria and the more potential for false-negatives.”

7) ***You have a lot of room to expand in the discussion section-why is ART important and why should additional studies be implemented for this population? Make a strong point here.***

The discussion has been substantially updated to better reflect the strength of research support for ART and more clearly discuss the path forward.

**Reviewer #2:**

***In the manuscript titled “Accelerated Resolution Therapy (ART) for the treatment of posttraumatic stress disorder in adults: A systematic study”, David et al firstly reported the efficacy of ART in the treatment of PTSD with a well-confirmed meta-analysis method. The authors found that there was a significant reduced of ART in PTSD symptoms from pre-post intervention. Although this is a well-established meta-analysis confirmed strictly in accordance with the guideline of Cochrane systematic reviews, some issues and some missed information need to be issued.***

1) ***The details about publications which was included or excluded from this study need to be supplied* [A]*, and a study flowchart need to be added in the revised manuscript* [B].**

**A)** This information can be found in supplementary material (S6 Appendix. Reasons for excluding studies or reports of studies during full text screening). I’m not sure if you have access to the supplementary material when reviewing. I have included a screen clipping of the appendix for your convenience:

**B)** The study flowchart is provided in Figure 1 after the first paragraph within the Results section (Fig. PRISMA 2020 Flow diagram of studies included in the review).

Note: the study flowchart image is in a separate file (Fig1.tif), so would not have been visible directly in the manuscript file. As per the PLOS Mental Health ‘Figures’ page under ‘How to Submit Figures and Captions’ ([Figures | PLOS Mental Health](https://journals.plos.org/mentalhealth/s/figures#loc-how-to-submit-figures-and-captions)): “Upload each figure as an individual file that is separate from the manuscript.” Below is a pasted copy of the Fig1.tif file with the figure caption and legend included below it:

**Fig 1. PRISMA 2020 flow diagram of studies included in the review.**

WHO = World Health Organization; ICTRP = International Clinical Trials Registry Platform; US VA = United States Department of Veterans Affairs; APA = American Psychological Association; ART = Accelerated Resolution Therapy.

2) ***Since only 11 related publications were included in the analysis, other potential database should be considered to add in screening the papers.***

As can be seen in the study flowchart above five separate electronic databases, three trial registers, and three websites were searched. Forward and backward citation searches were also conducted along with a search of the grey literature. The aforementioned searches were conducted on two separate occasions. Our search strategy was reviewed by a research librarian at our institutions medical library and meets criteria set out by the Peer Review of Electronic Search Strategies (PRESS). According to Charrois (2015): “The rule of thumb for a systematic review literature search is that more than 2 databases should be used.” We have significantly exceeded this minimum. Although I take your point that one can always search more databases, I feel that we have already reached the point of saturation. There really are very few studies on ART, and even fewer that met our eligibility criteria.

3) ***The tables, incl. table 1-2 lost some information due to the wrongly setting of output form.***

Thankyou for identifying this. Formatting of all tables in the manuscript have been corrected (e.g., the use of returns to align content has been replaced with the use of separate cells).

Note: if the table appears cut off when you are reviewing the manuscript you may have to switch from ‘Print View’ to ‘Draft View’. As per the PLOS Mental Health ‘Formatting Rules & Constraints’ ([Tables | PLOS Mental Health](https://journals.plos.org/mentalhealth/s/tables)): “Tables do not have strict width and height requirements. Do not split your table or otherwise try to make the table appear within the manuscript margins if it does not fit on one page. In Word, tables that run off of the manuscript page can be seen using Draft View. In the PDF version of the published article, very wide tables may be printed sideways, and long tables may span more than one page.”
